# Does a high threshold of sensory responsiveness affect the development of pretend play in children on the autism spectrum?

**DOI:** 10.1186/s11689-024-09551-y

**Published:** 2024-06-25

**Authors:** Karolina Krzysztofik

**Affiliations:** Department of Psychology of Occupation, Organization and Psychosocial Rehabilitation, Faculty of Social Science, John Paul II Catholic University of Lublin, Al. Racławickie 14, Lublin, 20-950 Poland

**Keywords:** Autism spectrum, Pretend play, Level of sensory responsiveness

## Abstract

**Background:**

Among the current avenues of research into the origins and development of the autism spectrum, those concerning atypical levels of sensory responsiveness are gaining increasing relevance. Researchers note the relationship of sensory responsiveness in children on the autism spectrum to their motor, cognitive and social development. Current research reports combines the responsiveness to sensory stimuli also with the development of pretend play. Aim of this study was to verify the relationship between the level of development of pretend play and the level of sensory responsiveness in children on the autism spectrum.

**Methods:**

A study was conducted in a group of 63 children with a diagnosis of autism spectrum aged from 3 years and 7 months to 9 years and 3 months using: *Pretend Play* subscale from the *Theory of Mind Mechanism Scale* and *Sensory Experiences Questionnaire version 2.1.*

**Results:**

The results revealed that elevated sensory hyporesponsiveness predicted low pretend play skills in the group of participating children.

**Conclusion:**

The study verified the contribution of the level of sensory hyporesponsiveness to explaining the atypical development of pretend play in children on the autism spectrum.

**Supplementary Information:**

The online version contains supplementary material available at 10.1186/s11689-024-09551-y.

## Introduction

There is something extraordinary about a child recreating in their play the scenarios of situations they have previously observed in everyday life. Many adults watch children’s play with intense attention and involvement. After all, play is not only a manifestation of hypothetical thinking, i.e. of the ability to handle and test possibilities, called by Gopnik [19] counterfactual reasoning, but also a manifestation of the emotions, desires, plans, anxieties, disappointments, hopes and a wide range of other emotional and mental experiences that the author of the game goes through. Watching a child at play is therefore an opportunity to gain an insight into their most intimate experiences. 

### Pretend play

In particular, the observation of the child’s symbolic play provides a rich picture of these experiences. The development of symbolic play begins as early as the first year of a child’s life and has two stages. The development of the first takes place between 1 and 4 years of age and involves the foundations of the ability to pretend in play [40]. It is a skill that encompasses a number of specific competencies: substitution (e.g. pretending that a building block is a telephone), attributing properties to objects (e.g. pretending that a teddy bear is sick) and the use of pretend scenarios (e.g. drinking tea from an empty cup, covering a teddy bear with a blanket, etc.) [22, 34]). Between the ages of 4 and 7, i.e. in the second stage of the development of symbolic play, it becomes subordinated to the roles assumed by those who play [40]. According to Leslie [34], pretend play is the relationship between three elements: the agent, the actual object, i.e. the primary representation, and a decoupled representation concerning the content of pretending. The process of decoupled representation is anchored in the *here and now* and is therefore temporal. It allows the child to attribute to an object or person characteristics that are distinct from the actual characteristics of that object or person. In this sense, the ability to pretend in play remains necessary in the development of symbolic play, which precedes the role play stage. Role play, on the other hand, gives a child a possibility to participate in imaginary social situations and to develop social skills [51].

### Pretend play in children on the autism spectrum

Children on the autism spectrum have been found to demonstrate a delay in the development of spontaneous pretend play manifestations compared to typically developing peers [47]. At the same time, a relationship between the level of development of pretend play and the severity of autism symptoms has also been observed [26, 31]. The delay in the development of pretend play skills is related to the atypical development of some cognitive and social skills observed in children on the autism spectrum: theory of mind [22, 34], praxis [35, 38] and playfulness [10].

### Pretend play and theory of mind

Contemporary authors [9, 22, 28, 34] agree that there is an interrelation between the development of pretend play skills and the development of the child’s theory of mind competence (understood as the ability to recognise the mental states of others). They emphasise the role of pretend play as a precursor [34], manifestation [22, 28] or correlate [9] of developing theory of mind. According to Leslie, the development of pretend play in a children precedes the development of their understanding of other people’s beliefs, true as well as false (1987). According to Baron-Cohen’s conception, skills such as, among others, joint attention and emotion recognition are early stages in the development of the theory of mind mechanism. At the same time, its most mature manifestation is the ability to understand false beliefs, which unfolds in typically developing children up to about four years of age [5, 6]. Hadwin and colleagues [22] and Kimhi [28] additionally include the ability to engage in pretend play within the range of manifestations of theory of mind but without specifying the relationship of this skill to theory of mind. Chan and colleagues emphasise the interdependence of the developmental pathways of pretend play and theory of mind (2016) [9]. Representatives of each of these three approaches (pretend play as a precursor, manifestation or correlate of the developing theory of mind) do not question the existence of an interdependence between pretend play and theory of mind, although their opinions as to the extent of this relationship differ.

The relationships between the development of different manifestations of theory of mind and sensory responsiveness to stimuli have been confirmed in a group of children on the autism spectrum. The studies revealed: an association between joint attention and hyperresponsiveness [4, 12]; an association between emotion recognition and reactivity to auditory, visual, tactile, gustatory and olfactory stimuli [16]; and an association between empathising and hyporesponsiveness [49]. All mentioned findings regard studied group of children aged 2 to 6 years [4, 12] or 6 to 11 years [16, 49]. However, there are no research findings confirming a relationship between pretend play and sensory responsiveness in a group of children on autism spectrum.

### Pretend play and motor planning skills

Motor planning is one of the most complex skills. It enables one to use new motor skills that are just being learned. Once these activities become automatic, they no longer require motor planning. This involves, among other things, a good body schema and the proper integration of sensations from the body’s own movement with tactile, visual and auditory stimuli. In fact, the development of praxis is determined by the course of sensory integration processes, including the level of responsiveness to sensory stimuli [2, 14].

Contemporary researchers emphasise that pretend play does not appear spontaneously in the development of children on the autism spectrum but requires prompting and demonstration by a teacher or parent [11]. The development of pretend play in those children, as well as in their typically developing peers, becomes significantly more effective when supported by parents [53]. It has previously been noted that children on the autism spectrum perform better in tasks requiring motor planning when they are provided with various (auditory, tactile, proprioceptive) stimuli than when they receive no additional stimulation at all [7]. For those children, pretend play is therefore a complex skill that, like any new motor activity in the early stages of development, requires practice and adult support.

It has been reported that difficulties in the development of praxis in children on the autism spectrum are related to a delay in the development of their social and symbolic play [35, 38]. At the same time, researchers [41] note that other sensory processing factors—body awareness, balance and touch—also contribute to the development of pretend play skills in typically developing children. In children on the autism spectrum, on the other hand it has been confirmed that difficulties in imitation praxis are related to the level of sensory responsiveness [43].Relationships have been pointed out between atypical development of imitation praxis and skills that, like pretend play, are manifestations of theory of mind: understanding false beliefs [39], joint attention [42], as well as emotion recognition [1].

### Pretend play and playfulness

The measures of playfulness are as follows: *Internal Locus of Control, Intrinsic Motivation, Suspension of Reality, Framing* [8]. *Intrinsic Motivation* involves such features in a child’s behaviour as engagement, play for play’s sake, not for its effect or reward, persistence and affect. *Internal Locus of Control* refers to two ranges of skills: in relation to self (making decisions, staying safe, modifying, interacting with objects) and in relation to others (negotiating, social play, supporting, initiating play, joining the play, participating). *Suspension of Reality* pertains to creativity in the use of objects and the ability to joke. *Framing* refers to the ability to give the clear play cues and to respond to the other’s cues, as well to maintain in the play theme [8].

However researchers [10] emphasise that the development of pretend play in this group of children is determined by only one measure of playfulness – *Internal Locus Of Control*. Other dimensions of playfulness are not associated with the development of pretend play in children on autism spectrum. It can therefore be concluded that pretend play only partially coincides in meaning with playfulness. Of note, none of the aforementioned measures of playfulness are dependent on the level of development of ideational praxis in children on the autism spectrum [37].

In sum, on the basis of existing research findings, it is not possible to make precise inferences about the relationship between pretend play and the level of responsiveness to sensory stimuli in children on autism spectrum.

## Methods

### Aim

The objective of the research conducted was to determine in what way the level of sensory responsiveness determines the level of development of pretend play in children on the autism spectrum. The main research hypothesis was formulated as follows.

H1. The development of pretend play in children on the autism spectrum is negatively determined by the level of sensory responsiveness.

The research findings to date confirm that abnormalities in sensory processing in children on the autism spectrum are related to their difficulties in skills that are manifestations of theory of mind, such as engaging in social play [38] or the ability to share attention [4, 12]. It was therefore hypothesised that also the level of development of pretend play would be related to the level of sensory responsiveness.

### Sample group

The criteria for the selection of the sample group were a diagnosis of autism spectrum and a level of speech comprehension corresponding at least to that of children aged 4 years. Before proceeding to assessing pretend play, the level of speech comprehension was assessed using the Speech Comprehension subscale of the IDS-P test [21] in a Polish translation and adaptation [17]. The recruitment of children for the study was conducted by contacting the management staff of the educational or therapeutic facilities they attended. Ultimately, the study involved a group of 63 children with a diagnosis of autism spectrum, aged from 3 years and 7 months to 9 years and 3 months. The mean age of participants was 5 years and 9 months (SD = 1.59). The largest subgroup was children aged from 7 years to 9 years and 3 months (24 children, representing 38.09% of all participating children). The smallest group were children aged from 3 years and 7 months to 4 years and 11 months (13 children, representing 20.64% of participants). Of the participating children, 34.93% were aged from 5 years to 6 years and 11 months. For four children, no information on age was obtained (the parent did not specify the exact age of the child in years and months). A disproportion in the size of the subgroups of girls (17.5%, 11 participants) and boys (82.5%, 52 participants) was observed. Surveyed children were residents of medium-sized and large cities (30.16% and 23.80% of participants, respectively), rural areas (28.58% of participants) and small towns (12.70% of participants). For three children, no information was obtained on their place of residence. The participants were educated in: inclusive kindergartens (79.37%, 50 participants), kindergartens for children with special educational needs (7.93%, 5 participants), schools for children with special educational needs (6.34%, 4 participants), and inclusive schools (3.18%, 2 participants). One child attended a therapeutic school (1.59%) and another a special care educational facility (1.59%). The sample group had diagnoses of autism, infantile or early childhood autism (87.31%). Some of the children also had diagnoses of Asperger Syndrome (7.93%) and atypical autism (4.76%).

### Research tools

The study reported in this article used the Sensory Experiences Questionnaire version 2.1 (SEQ) by Baranek [3], translated into Polish by [30] – measuring the level of sensory responsiveness, the Pretend Play subscale from the Theory of Mind Mechanism Scale (SToMM) (2016) to assess the level of development of pretend play and also a sociodemographic datasheet.

The sociodemographic datasheet contained questions to the child’s parent/caregiver concerning: the type of diagnosis, the presence of comorbid conditions, the type of educational establishment the child attends and the family’s place of residence. Parents were also asked to provide in the questionnaire the detailed name of their child’s diagnosis (e.g. early childhood autism, autism spectrum, atypical autism, Asperger’s syndrome) exactly as it appears in their child’s medical file.

*The Sensory Experiences Questionnaire version 2.1 (SEQ)* by Baranek [3] translated into Polish by [30] (translation consultation by M. Wiśniewska, PhD) is used for measuring the level of sensory responsiveness in children on autism spectrum. The results of this tool are as follows: a total score and specific scores for patterns of sensory responsiveness (Hyporesponsiveness – HYPO, Hyperresponsiveness – HYPER, Sensory seeking – SEEK). The results of the subscales are correlated to each other. There are some other scores regarding the sensory modality categories (Auditory – AUD, Visual – VIS, Tactile – TACT, Gustatory and Olfactory – GUST AND OLF, Vestibular and Proprioceptive – VEST and PRO) and regarding the sensory context (Social Context – SOCIAL, Nonsocial Context – NONSOCIAL). In the present study, it was decided that all the results obtained by the children would be analysed in order to gain as detailed a picture as possible of the relationship of pretend play with sensory responsiveness. The child’s parent/caregiver provides answers on a 5-point Likert scale. Higher scores represent more severe sensory responsiveness. Cronbach’s α coefficients showed satisfactory values for the total score (0.83) and for most of individual dimensions (HYPER: 0.81; HYPO: 0.67; SEEK: 0.78; SOCIAL: 0.61; NONSOCIAL: 0.80) and modalities (VIS: 0.64; TACT: 0.66). However, three subscales showed insufficient values (AUD: 0.53; VEST and PRO: 0.23; GUST AND OLF: 0.43).

*The Theory of Mind Mechanism Scale (SToMM)* [29] is based on the concept and educational program of mind-reading skills by P. Howlin, S. Baron-Cohen and J. Hadwin [25]. This tool makes it possible to assess the level of development of the elements of the *Theory of Mind Mechanism*: emotion recognition, understanding beliefs and ability to pretend play. The presented study used the Pretend Play subscale (SToMM_PP). During the experiment, the child’s free play with a set of toys (a toy cleaning set, a toy DIY set, a large rag doll “Krzyś”, a toy cup and teaspoon, a toy house with furniture and two lego figures: a boy and a girl) is observed during 10 min of spontaneous, nondirected play. The child may also choose to play with other toys available in the room. The child is encouraged by the researcher to play freely, and for the levels of pretend play (4 and 5) some simple additional scenarios are dictated by the researcher (e.g. brushing teeth), and then additional questions are asked of the child to assess whether he/she can distinguish pretending from reality (e.g. the researcher asks the child who is pretending to brush teeth with an imaginary toothbrush, “Are you really brushing your teeth or are you pretending?”). The answers provided by the child to these questions make it possible to assess whether he or she can distinguish between pretending and reality in three areas: substitution of an object, pretend action and pretend scenario. This information is additional to assessing the level of development of pretend play. Observing in what way and how many times the child uses the toys (for sensorimotor, functional or pretend play) makes it possible to assign the child to one of the levels of development of pretend play skills: no development of sensorimotor play, sensorimotor play (non-specific: shaking, smelling, tasting, sorting, etc.), emerging functional play (1 or 2 examples of functional play, e.g. putting a spoon in a cup, pushing a car, etc.), established functional play (at least of 3 examples of functional play), emerging pretend play (1 or 2 examples relating to: substitution of objects, e.g. a building block representing a car, attribution of characteristics, e.g. pretending a teddy bear has a dirty face, use of imaginary scenarios, e.g. drinking from an empty cup), established pretend play (minimum of 3 examples in the following areas: substitution of objects, attribution of characteristics and use of imaginary scenarios). If a child refuses to participate or participates for a very short time (e.g. 2–3 min) without showing any signs of pretend play, his/her participation is not scored. A pilot study was conducted with 26 children on the autism spectrum and 25 children with typical development between the ages of 4 years 4 months and 8 years 11 months. All of the typically developing children attained development of pretend play at level 5, while the children on the autism spectrum had a mean score of 4.23 (SD = 1.17). These were statistically significant differences [32]. The tool was not validated in children with neurodevelopmental diagnoses. Cronbach’s α for this subscale is 0.78.

### Procedure

Parents or caregivers of the participating children read detailed written information about the conditions of the study. After expressing written consent for participation, the parents/caregivers were asked to complete the *Sensory Experiences Questionnaire version 2.1* and the sociodemographic datasheet.

The participating children were invited to individual sessions during which their level of development of pretend play was assessed. The sessions took the form of play and were held in a room familiar to the children in the educational or therapeutic facility attended by the child. All of them lasted approximately 10 min. In most cases, during the assessment, the researcher was alone with the participating child. In a few cases, a support teacher was present. It was left for the teacher to decide whether the teacher would be present during the assessment. The teacher was asked not to assist the child in organising play or answering the researcher’s questions. At the end of the session, every child received a tiny toy (a yo-yo, a sensory ball, a squeeze toy etc.) as an expression of gratitude for the participation.

IBM SPSS 25 version with the PROCESS extension [24] was used for the statistical analysis. For statistical analyses, linear regression analysis in the stepwise model was used to verify the hypothesis. All sensory responsiveness level variables were included in one model. Stepwise regression allows only those variables that are significant predictors of the dependent variable to be entered into the model. Variables that are not significant are eliminated. Also, the successive inclusion of predictors in the stepwise regression makes it possible to control for their mutual co-variance. Additionally, Student’s t-test was used to verify differences in terms of the level of sensory responsiveness in subgroups of children who scored low and high on the development of pretend play.

The research project was approved by the Research Ethics Committee Institute of Psychology at the John Paul II Catholic University of Lublin, Poland.

## Results

The presentation of the results begins with a description of the state of the variables analysed – the level of sensory responsiveness and the level of pretend play in the group of participating children (Table 1).

**Table 1:** Sensory responsiveness level and level of pretend play in a sample of studied children

		M	SD
SToMM	PP	4.35	1.19
SEQ	TS	2.27	0.50
	HYPER	2.15	0.66
	HYPO	1.91	0.70
	SEEK	2.47	0.66
	SOCIAL	2.11	0.56
	NONSOCIAL	2.33	0.54
	VIS	2.24	0.67
	AUD	2.21	0.75
	TACT	1.98	0.59
	VEST and PRO	2.66	0.69
	GUST and OLF	2.40	0.68

*Note SToMM_PP* – Pretend Play subscale from the *Theory of Mind; Mechanism Scale; SEQ* – Sensory Experiences Questionnaire; *TS* – total score; and scores in the dimensions of: *HYPER* – Hyperresponsiveness; *HYPO* – Hyporesponsiveness, *SEEK* – Sensory seeking; *SOCIAL* – Social context, *NONSOCIAL* – Nonsocial context and in the modalities: *VIS* – visual, *AUD* – Auditory, *TACT* – Tactile; *VEST and PRO* – Vestibular and proprioceptive, *GUST and OLF* – Gustatory and olfactory

In terms of the level of general responsiveness, as well as in terms of the level of responsiveness in particular dimensions and modalities, the results obtained by participants oscillate between 1.91 and 2.66. These values are similar to the theoretical mean for the tool used (*Sensory Experiences Questionnaire version 2.1*). On their basis, it is possible to draw conclusions about the average level of sensory responsiveness of the participating children. They obtained the highest results in modalities concerning vestibular and proprioceptive stimuli, gustation and olfaction, as well as in the dimension of sensory seeking. The lowest results obtained by participating children were in the dimension of hyporesponsiveness to sensory stimuli and the tactile modality (Table 1).

The mean score for the development of pretend play skills in the children was 4.35, which corresponds to level 5 of its development, i.e. established pretend play (Table 1).

Significant model of the relationship between the level of development of pretend play and the level of sensory responsiveness are presented below (Table 2).

**Table 2:** Regressions for pretend play level

Pretend play level				
*R*^*2*^ = 0.17, *F* = 12.68 *p* < .001				
	*ß*	*t*	*p*	*95%CI*
Constant	5.68 (B)	14.21	*p* < .001	4.88:6.48
Sensory hyporesponsiveness	− 0.41	-3.56	*p* < .001	-1.09:-0.30

*Note R*^**2**^—model fit coefficient; *t—*test statistic; *β*—standardised regression coefficient; *p—*statistical significance

For a more detailed analysis of the relationship of pretend play with the level of sensory responsiveness in the participating children, it was decided that the level of responsiveness would be compared in two subgroups of children: those who scored low (3 and below – no manifestation of pretend play) on the Pretend Play subscale of the SToMM and those who scored high (4 and 5 – presence of pretend play) on that Subscale. The results of the analyses indicate that the subgroup of children with low scores on pretend play have higher levels of sensory hyporesponsiveness, as well as responsiveness in the tactile modality and in the nonsocial context, than the subgroup of children with high levels of pretend play development. Furthermore, differences between these subgroups in responsiveness in the visual modality and in the social context are on the borderline of significance (Table 3).

**Table 3:** Sensory responsiveness level in groups of children with low and high scores in pretend play

	SToMM_PP > 3 *N* = 52	SToMM_PP ≤ 3 *N* = 11				
SEQ	M (SD)	M (SD)	df	t	p	d
TS	2.23 (0.53)	2.42 (0.37)	61	1.68	0.097	
HYPER	2.12 (0.67)	2.25 (0.64)	61	1.15	0.127	
HYPO	1.78 (0.61)	2.40 (0.85)	61	3.35	0.001	0.65
SEEK	2.46 (0.67)	2.54 (0.64)	61	0.67	0.504	
SOCIAL	2.05 (0.57)	2.34 (0.43)	61	2.03	0.046	0.54
NONSOCIAL	2.29 (0.57)	2.45 (0.36)	61	2.29	0.029	0.53
VIS	2.13 (0.75)	2.53 (0.67)	61	2.06	0.043	0.73
AUD	2.16 (0.70)	2.51 (0.42)	61	1.62	0.109	
TACT	1.90 (0.60)	2.25 (0.49)	61	2.61	0.011	0.57
VEST and PRO	2.65 (0.73)	2.67 (0.54)	61	0.89	0.381	
GUST and OLF	2.44 (0.69)	2.24 (0.62)	61	0.54	0.589	

*Note SToMM_PP > 3* – subgroup of children who scored in Pretend Play subscale from the *Theory of Mind Mechanism Scale* 3 or less; *SToMM_PP ≤ 3* – subgroup of children who scored in Pretend Play subscale from the *Theory of Mind Mechanism Scale* 4 or 5; *t*—test statistic; *df*—the number of degrees of freedom; *d*—Cohen’s d

## Discussion

The author’s research partially verifies the hypothesis that the development of pretend play in a group of children on the autism spectrum is determined by the level of responsiveness to sensory stimuli. It reveals that, of the many dimensions and modalities of sensory responsiveness, only the level of hyporesponsiveness is a predictor for the level of development of pretend play in the group of children on the autism spectrum. Therefore, a high threshold for responding to stimuli of different modalities determines a weaker development of pretend play in children on the autism spectrum. Such findings are consistent with analyses by [38] indicating a link between social play (including pretend social play) and the development of praxis in this group of individuals. The author’s research complements these findings by indicating that difficulties in pretend play (also of a social nature) are related to a high threshold for responding to sensory stimuli. A high stimulus-response threshold (sensory hyporesponsiveness) is associated with difficulties in perceiving stimuli and focusing attention on them [2]. May therefore be an obstacle to perceiving and learning the possible uses of toys and objects in pretend play and to planning a scenario for playing with them.

At the same time, it is worth noting that other measures of sensory responsiveness (including sensory hyperresponsiveness and sensory seeking) proved to be non-significant predictors for the level of pretend play in the sample group of children. At this point, it is worth mentioning that the sample group was characterized by little heterogeneity in the scores obtained for the level of development of pretend play. The majority of the group scored high (4 or 5) which also resulted in high scores when converted to mean scores (M = 4.35; SD = 1.19) (Table 1). Possibly, in children who perform well in pretend play, sensory hyperresponsiveness and sensory seeking are not as important for the development of pretend play as sensory hyporesponsiveness.

Additional analyses indicating differences between subgroups with high and low scores in pretend play suggest that hyporesponsiveness to tactile stimuli and to the stimuli present outside the social context may predict the pretend play level in children on the autism spectrum. Abnormal levels of responsiveness to tactile stimuli, on the other hand, are associated with difficulties in praxis [2]. This leads to the assumption that praxis is involved in the relationship between sensory hyporesponsiveness and pretend play in children on the autism spectrum. Confirmation of such an assumption in future studies would be substantial contribution to the existing evidence indicating the association of the development of a child’s praxis with symbolic and social play skills [35]. The proven relationship between social and symbolic play and praxis [35, 38] in children on autism spectrum suggests to measure also the other motor skills, such as coordination in future research.

Apart from little heterogeneity in the scores obtained for the level of development of pretend play for the sample group, another limitation of the analyses conducted in this study is the lack of a control group of typically developing children. It would be advisable to repeat the study using the analyses in the control group, and ensuring that a group of children on the autism spectrum with more heterogeneous levels of development of pretend play is selected. In this way, the results obtained and the conclusions drawn will be better substantiated. Among the directions for future analysis, it is also worth mentioning the study of the role of pretend play in the relationship of sensory responsiveness with the severity of autism symptoms. Current research evidence reveals an association of autism symptom severity in terms of communication and interaction and restricted patterns of interests and activities with sensory responsiveness [23, 33, 44, 45, 54] as well as with pretend play [26, 31]. They therefore lead to the conclusion that pretend play may play role in the relationship between the level of sensory responsiveness and the severity of autism symptoms. It also seems worthwhile to repeat the study while controlling for the variables of age, level of communication and language development (including speech comprehension) in the participating children, which have been suggested by researchers to have a significant impact on the development of pretend play in typically developing children [36, 50] as well as in children on the autism spectrum [10]. Moreover, the level of speech development is related with the severity of autism symptoms [52]. The strength of the relationship between sensory responsiveness and the severity of autism symptoms may therefore differ in children with different levels of speech and communication development. Another important future research direction may be to examine the associations among pretend play, sensory responsiveness and skills such as praxis and theory of mind. Only in a few cases a support teacher was present during the session with the child. Therefore, it is difficult to assess the real significance of the teacher’s presence for the child’s performance in pretend play or sensory responsiveness level. It is worth to control this variable in the future research. Including the presence of a support teacher in the model explaining pretend play may provide important information on the role of presence of other people for the pretend play in children on the autism spectrum.

## Conclusions

The partially verified hypothesis that the development of pretend play in a group of children on the autism spectrum can be predicted by the level of responsiveness to sensory stimuli indicates that difficulties in pretend play are related to a high threshold for responding to sensory stimuli. High stimulus-response threshold (sensory hyporesponsiveness) is associated with difficulties in perceiving stimuli and focusing attention on them [2], and may therefore be an obstacle to perceiving and learning the possible uses of toys and objects in pretend play and to planning a scenario for playing with them. The proven differences between subgroups with high and low scores in pretend play suggest that hyporesponsiveness to tactile stimuli and to the stimuli present outside the social context may be a particularly important predictor of the development of pretend play in children on the autism spectrum.

The verified role of the level of hyporesponsiveness to sensory stimuli in the development of pretend play in children on the autism spectrum may have implications for the practice of rehabilitation of this group of children. This is because reducing high levels of hyporesponsiveness to sensory stimuli may play some role in supporting the development of pretend play in this group of children.

### Electronic supplementary material

Below is the link to the electronic supplementary material.

Supplementary Material 1

Supplementary Material 2

## Data Availability

Not applicable.
