# Supplementary material for: Does a high threshold of sensory responsiveness affect the development of pretend play in children on the autism spectrum?
Source: J Neurodev Disord. 2024 Jun 25;16:34. doi: 10.1186/s11689-024-09551-y (PMC11197220; doi:10.1186/s11689-024-09551-y)
Supplement: Supplementary file 1 — Supplementary Material 1 [file 11689_2024_9551_MOESM1_ESM.pdf]

Dear Sir/Madame,

Thank you for preparing another review of the manuscript entitled *Does a High Threshold of Sensory Responsiveness Affect the Development of Pretend Play in Children With Autism Spectrum Disorder?* I listed changes that were made in the text according to the comments.

It is difficult to determine the importance of the presence of a support teacher for the children's performance, as the teacher was present during the session only in a few cases (four-five). Therefore, it was decided not to include such variable in the current model. The inclusion of the teacher's presence during the session as an important variable in the model was indicated as recommendations for the future research.

The Cohen's d values in the Table 3 were rechecked. The obtained results were the same as in the previous calculations. I understand that in places where the differences were not significant, the Cohen’s d values could be confusing to the reader. Therefore, I decided to remove them and leave them only in places where the differences were significant.

Thank you for all the comments.

Respectfully,
