# Supplementary material for: Does a high threshold of sensory responsiveness affect the development of pretend play in children on the autism spectrum?
Source: J Neurodev Disord. 2024 Jun 25;16:34. doi: 10.1186/s11689-024-09551-y (PMC11197220; doi:10.1186/s11689-024-09551-y)
Supplement: Supplementary file 2 — Supplementary Material 2 [file 11689_2024_9551_MOESM2_ESM.pdf]

Dear Sir/Madame,

Thank you for preparing another review of the manuscript entitled *Does a High Threshold of Sensory Responsiveness Affect the Development of Pretend Play in Children With Autism Spectrum Disorder?* Below I listed changes that were made in the text according to the comments.

The Table 3 heading was changed to the shorter version.

The reasons for the decision to use the form *children on the autism spectrum* instead of *autistic children* were justified with a reference to literature.

The linguistic side of the text was consulted with two independent interpreters. Some sentences were shortened. It was difficult for me and for the interpreters to find any grammar shortcuts as you haven't indicate the places were grammar improvement were needed. I'm sorry if changes are insufficient.

Thank you for all the comments.

Respectfully,
